# Effect of Intravascular Ultrasonography (IVUS) Imaging on Stent Optimization in Patients Achieving Optimal Results After Angiographic-Guided Stent Implantation: A Non-randomized Interventional Study

**DOI:** 10.7759/cureus.77458

**Published:** 2025-01-15

**Authors:** Pankaj Kumar Singh

**Affiliations:** 1 Department of Cardiology, All India Institute of Medical Sciences, Rishikesh, Rishikesh, IND

**Keywords:** coronary angiography, double vessel disease, intravascular ultrasound, left main coronary artery, optical coherence tomography, single vessel disease, stent thrombosis, target lesion revascularization, target vessel revascularization, triple vessel disease

## Abstract

Background: Angiography possesses limitations in assessing plaque composition, vessel width, diffuse reference vessel disease, lesion severity, and the success or failure of stent placement. Intravascular ultrasonography (IVUS) helps address several of these issues by offering enhanced visualization of the coronary architecture and stent placement.

Methods: This study was conducted at the Department of Cardiology of All India Institute of Medical Sciences, Rishikesh. It spanned 12 months with a six-month follow-up and involved post-angiography-guided angioplasty and stenting. All patients underwent IVUS pullback. Due to the non-working of the unspecified surgical package under the Ayushman Bharat scheme, the study included 16 patients, with an intended target of 30 patients.

Results: Half of the patients (eight, 50%) presented with acute coronary syndrome, while the other half presented with chronic stable angina. Left main coronary artery disease was present in 50% of patients, and 13 (81.25%) had complex coronary artery lesions. After angiography-guided angioplasty, IVUS pullback on all patients revealed that only three patients (18.75%) required post-IVUS optimization of the stent.

Conclusion: The majority of patients did not require post-IVUS optimization following angiography-guided angioplasty and stenting. No major adverse cardiovascular events were recorded, and no target vessel myocardial infarction (MI), target vessel revascularization, target lesion revascularization, or stent thrombosis (ST) were reported over the six-month follow-up period.

## Introduction

Coronary angiography is the standard technique used in diagnosing coronary artery disease and in guiding angioplasty. This technique, however, is inherently limited because it only projects in two dimensions. Limitations of this method include difficulties in assessing the composition of plaques, vessel dimensions, diffuse reference vessel diseases, lesion severity, and success or failure of stent deployment. Over the last 30 years, intravascular ultrasonography (IVUS) has gained increasing prominence in clinical practice. IVUS resolves several of the problems inherent in coronary angiography by providing more vivid images of the architecture of the coronary arteries and stent positioning. IVUS creates high-resolution tomographic images of the arterial vessel in real time [1-3].

More information is provided by intravascular imaging techniques, including optical coherence tomography (OCT), IVUS, and coronary angiography (CA), compared with traditional radiographic coronary angiography. Drug-eluting stents (DESs) are increasingly used in percutaneous coronary intervention (PCI) due to their efficiency in preventing restenosis and revascularization requirements. Nevertheless, problems like stent thrombosis (ST) and restenosis remain, mostly in complex lesions, associated with higher mortality. Under expansion, residual edge disease, and dissections are some of the suboptimal stent placement that affect future event rates [1-3]. Observational studies and randomized controlled trials have shown IVUS guidance to be effective in PCI. However, IVUS-guided PCI has not been adopted into routine practice because of additional costs, longer procedure times, and no significant decrement in cardiac mortality.

This study is to assess patients with coronary artery disease who underwent traditional angiography-guided angioplasty followed by IVUS imaging. The study will assess the changes in minimum lumen diameter and minimal lumen area pre- and post-procedure and determine the percentage of patients with stent malapposition following angiography-guided coronary angioplasty and stenting. Furthermore, it monitors ST, revascularization of the target lesion, revascularization of the target vessel, and cardiovascular events, which are adverse (MACE) for six months post-procedure.

## Materials and methods

Population and study design

This study adopts a non-randomized interventional approach conducted at the Department of Cardiology, All India Institute of Medical Sciences, Rishikesh over 12 months after obtaining approval from the Institute Research and Ethics Committee (AIIMS/IEC/22/651), with subsequent six-month follow-up. The study includes patients suffering from coronary artery disease who are admitted under the Department of Cardiology, AIIMS Rishikesh, and undergoing invasive coronary angiography. Eligibility criteria include patients >18 or more years of age with unstable angina, chronic stable angina myocardial infarction (MI) without or with an elevation of the ST segment (more than 24 hours post-onset of chest discomfort), and new coronary lesions suitable for DES implantation.

Procedural overview

The operator selects either the radial or femoral approach for coronary angiography and angioplasty. The principal investigator and the guide assess the coronary artery disease on angiography to minimize subjective errors in interpretation. All eligible patients undergo coronary angioplasty and stenting followed by IVUS imaging to detect stent malapposition based on the IVUS criteria. This involves calculating the minimal luminal diameter of the stented segment and the minimal luminal area of the stented segment, optimizing the stent as per IVUS guidance (based on stent under-sizing or stent mal apposition). In addition, IVUS imaging post-procedure is performed (if needed). This study uses the Philips Volcano CORETM Series s5 imaging system with a Philips Eagle Eye Platinum ST digital IVUS catheter (Philips, Amsterdam, Netherlands), which has a resolution of 20 MHz.

Definitions and endpoints

Minimum lumen diameter (MLD) is the smallest diameter through the center point of the lumen [4]. Minimum lumen area (MLA) is the smallest area through the center point of the lumen [4]. MACE (major adverse cardiac event) is a composite of total death, MI, stroke, hospitalization due to heart failure, and revascularization, including PCI and CABG [5-7].

Target lesion revascularization (TLR) is any repeat percutaneous intervention of the target lesion or bypass surgery of the target vessel performed for restenosis or other complications of the target lesion. In this case, the definition of the target lesion can be referred to as being the treated segment from 5 mm proximal to the stent to 5 mm distal to the stent [8-9]. TVR (target vessel revascularization) is a clinically driven - as defined for TLR - repeat percutaneous intervention of the target vessel or bypass surgery of the target vessel, recurrent Q-wave or non-Q-wave MI, or cardiac death not clearly ascribable to a vessel other than the target vessel [8-9].

Stent thrombosis (ST) is defined as either diagnosed by angiographic or pathologic confirmation. This study adopts the standard definitions proposed by the Academic Research Consortium (ARC) [10].

Angiographic documentation of ST is the presence of a thrombus (intracoronary thrombus) that occurs in the stent or in the segment 5 mm proximal or distal to the stent and at least one of the following criteria that occur within a 48-hour time window: a) new onset of ischemic symptoms at rest; b) new ischemic ECG changes that suggest acute ischemia; and c) typical rise and fall in cardiac biomarkers (see definition of spontaneous MI) [11-14].

Pathological confirmation of ST is evidence of a recent thrombus within the stent at autopsy or through examination of tissue obtained after thrombectomy.

Primary Objective

The primary objective of this study is to estimate the proportions of patients with stent malapposition on IVUS imaging who have achieved optimal results after angiography-guided stent implantation.

Secondary Objectives

The secondary objectives of this study are to determine the difference between the final minimum lumen diameter and final minimal lumen area following IVUS imaging of the stented segment, compared to the angiography-guided minimal lumen diameter and minimal lumen area of the stented segment; to assess the incidence of major adverse cardiovascular events (MACEs) that occurred over six months; and to assess the occurrence of target vessel MI, target vessel revascularization, target lesion revascularization, and stenting over a six-month follow-up period in patients who receive IVUS-guided coronary angioplasty and stenting.

Statistical analysis

The study utilizes an expert statistical program IBM SPSS Statistics for Windows, Version 23.0 (released 2015, IBM Corp., Armonk, NY) to analyze the data. Categorical data are presented as percentages and proportions, and numerical values are reported as mean ± standard deviation (SD).

## Results

The study commenced from December 2022 to January 2023, followed by a six-month follow-up. Due to financial issues from the patient side and issues related to the Ayushman Bharat scheme's U-100 package, it did not work for the last seven to eight months. Accordingly, the study could not provide the benefit of the unspecified surgical package (U-100 package) in patients undergoing IVUS. Out of 16 patients, 12 patients (75%) were from Uttarakhand, and the remaining four patients were from Uttar Pradesh. Twelve (75%) patients were male, and only four were female. Eight patients (50%) presented with acute coronary syndrome, and the other half (eight, 50%) presented with chronic stable angina with the youngest patient at 41 years. Notably, five individuals (31.25%) had hypertension, four (25%) had diabetes, and five (31.25%) were smokers. Additional baseline characteristics are summarized in Table 1.

**Table 1:** Baseline characteristics LVEF: left ventricular ejection fraction, ACEi: angiotensin-converting enzyme inhibitor, ARB: angiotensin receptor blocker, MRA: mineralocorticoid receptor antagonist, CCS: chronic coronary syndrome, STEMI: ST-segment elevation myocardial infarction, NSTEMI: non-ST-segment elevation myocardial infarction

Age --- years	56 +/- 8
Male sex --- no. (%)	12 (75%)
Hypertension ---- no. (%)	5 (31.25%)
Diabetes ---- no. (%)	4 (25%)
Dyslipidemia --- no. (%)	1 (6.25%)
Smoking --- no. (%)	5 (31.25%)
LVEF ---- no. (%)	
Normal	7 (43.75%)
Mild impaired (>40 %)	4 (25%)
Moderate impaired (<40 %)	5 (31.25%)
CCS --- no. (%)	8 (50%)
Unstable angina --- no. (%)	2 (12.5%)
NSTEMI ---- no. (%)	1 (6.25%)
STEMI --- no. (%)	5 (31.25%)
Antiplatelet given on discharge no. (%)	
Aspirin	16 (100%)
Ticagrelor	15 (93.75%)
Clopidogrel	1 (6.25%)
Statin --- no. (%)	16 (100%)
Beta-blocker --- no. (%)	16 (100%)
ACEi/ARB --- no. (%)	11 (68.75%)
SGLT2 I	11 (68.75%)
MRA	6 (37.5%)

The echocardiographic findings indicated that five patients (31.25%) had anterior wall hypokinesia, two (12.5%) had inferior wall hypokinesia, two had global LV hypokinesia, and seven (43.75%) had normal left ventricular ejection fraction (LVEF).

Regarding the procedural details, eight patients (50%) had left main coronary artery disease and 13 (81.25%) had complex coronary artery lesions. Eight patients (50%) underwent percutaneous transluminal coronary angioplasty (PTCA) using three drug-eluting stents (DESs), three patients underwent PTCA using one DES, four patients underwent PTCA using two DESs, and one patient underwent PTCA using four DESs. Ten patients (62.5%) underwent PTCA to LAD, two patients (12.5%) underwent PTCA to LCX, and two patients (12.5%) underwent PTCA to RCA. The maximum stent diameter in LMCA was 4 mm. The distribution of stents used and the vessels treated are detailed in Table 2*.*

**Table 2:** Procedural characteristics LM: left main, SVD: single vessel disease, DVD: double vessel disease, TVD: triple vessel disease, DES: drug-eluting stent, IVUS: intravascular ultrasonography

Patients with lesions --- no. (%)	
SVD	3 (18.75%)
DVD	4 (25%)
TVD	1 (6.25%)
LM	8 (50%)
LM + SVD	0
LM + DVD	5 (31.25%)
LM + TVD	3 (18.75%)
Syntax Score 1	21.719 +/- 11.32
No. of stents --- no. (%)	
1 DES	3 (18.8%)
2 DES	4 (25%)
3 DES	8 (50%)
4 DES	1 (6.2)

## Discussion

This study found that most patients presented with complex coronary artery lesions, yet only a small fraction, specifically three individuals (18.75%), needed post-IVUS optimization of their stents. Most patients underwent angiography-guided angioplasty and stenting without requiring post-IVUS optimization. The IVUS pullback examination confirmed the appropriate stent positioning and no notable adverse cardiovascular incidents were detected. Over the six-month follow-up period, there were no occurrences of myocardial infarction in the specific coronary artery, no need for additional medical intervention in the specific coronary vessel, or additional medical intervention in the specific lesion or stent occlusion. 

This study had many limitations: 1) Incorporating intravascular imaging into the PCI treatment increases the overall cost, in contrast to using angiography alone. Due to the ineffectiveness of the unspecified surgical package (U-100 package) inside the Ayushman Bharat scheme, we were unable to reach the intended number of patients. 2. It is a single-center data and the study was non-randomized. 3. Longer follow-up of these patients might have affected the final outcome.

Two recent randomized studies, IVUS-XPL and ULTIMATE, were included in a meta-analysis by Hong et al. [15-18]. The study's objective was to compare the two PCI techniques in 2,577 patients who had the operation with 28 mm or longer second-generation DESs. The results indicated that IVUS-guided PCI significantly reduced cardiovascular mortality, myocardial infarction, and ST throughout a three-year follow-up period. Patients who fulfilled the IVUS criteria for optimum stent implantation had a substantially reduced risk of ST, MI, and cardiac death compared to those who did not.

A five-year follow-up from the IVUS-XPL study further demonstrated that for lengthy lesions treated with Xience Prime stents, IVUS-guided PCI significantly lowered the incidence of serious adverse cardiovascular events compared to angiography-guided PCI. Unlike angiography-guided PCI, IVUS-guided PCI substantially decreased target artery failure and ST at the three-year follow-up, according to the ULTIMATE third study.

The large-scale randomized controlled trial called OPTICUS aimed to determine whether IVUS could reduce the occurrence of in-stent restenosis [19]. This study is significant since it is the largest of its kind, and it aimed to find the in-stent restenosis rate, lowest blood vessel diameter, and percentage of narrowing at the six-month follow-up. As a secondary objective, it aimed to determine the frequency of major adverse cardiovascular events at six and 12 months of follow-up. Since the two groups did not vary significantly in any of the goals, the study concluded that IVUS is not beneficial for stenting.

The TULIP trial compared IVUS with angiography for guiding stent implantation in lesions longer than 20 mm [20]. The trial identified positive results in patients with a high risk of in-stent restenosis who received IVUS monitoring. However, these studies found no evidence that routine IVUS during stenting procedures reduced the occurrence of in-stent restenosis.

## Conclusions

The cardiovascular team at the All India Institute of Medical Sciences, Rishikesh, conducted this study as a non-randomized interventional trial. Beginning in December 2022 and concluding in January 2024, the research spanned a full calendar year followed by six months of patient follow-up. This study’s primary aim was to evaluate the role of IVUS in ensuring optimal stent placement post-angiography, and the results suggest that while IVUS may not always be necessary for immediate optimization, it plays a crucial role in complex cases.

The findings indicate that while most patients had complex coronary artery lesions, only a minority of three individuals needed IVUS-guided stent optimization. Most patients underwent angiography-guided angioplasty and stenting without requiring post-IVUS optimization. The IVUS pullback examination confirmed the appropriate positioning of the stent, and no notable adverse cardiovascular incidents were detected. There were no occurrences of MI in the specific coronary artery, need for more medical intervention in the specific coronary vessel, or necessity for additional medical intervention in the specific lesion or stent occlusion documented during the six-month post-treatment monitoring period.
